# Engineering *Thermobifida fusca* cutinase variants for the sustainable recycling of PET from cotton-blended textiles

**DOI:** 10.3389/fsysb.2026.1914536

**Published:** 2026-09-08

**Authors:** Neo Su, Dylan Bo Huang, Tzu-Chun Pu, Ian Cheng, Pin-Hua Chen, Chi-Cheng Hsieh, YuTing Lin, Chi Hou Ng, Ya-Shan Yu, Jakie Ting, Yu-Chuen Lai, Jason Wang

**Affiliations:** GEMS Academy, Taipei City, Taiwan

**Keywords:** blend textile degradation, enzyme engineering, mutagenesis, polyethylene terephthalate (PET), Thermobifida fusca cutinase

## Abstract

**Introduction:**

Recycling polyethylene terephthalate (PET) from cotton-blended fabrics is constrained by substrate access. Textile PET is more crystalline and occluded than the amorphous film on which most PET hydrolases are optimized, and whether engineered enzymes retain their advantage as substrate complexity rises remains untested.

**Methods:**

Six *Thermobifida fusca cutinase* 2 (*Tf*Cut2) variants were designed by structure-guided modelling and machine-learning prediction. Activity was screened with p-nitrophenyl butyrate (pNPB) and on PET film across 35–70 °C and pH 7.0–8.5, then assayed on pure PET fiber, knit and mesh textiles, and cotton–PET blends, with and without pretreatment in 15% NaOH at 80 °C for 4 h. Degradation was quantified as absorbance at 260 nm (A260), a relative measure of soluble product release.

**Results:**

Under a unified condition (51.8 °C, pH 7.5), five variants were active on PET film, and MC-nc27 was inactive throughout. Advantage over wild-type widened with substrate complexity, reaching 14.3-fold for A65H/L90A/I213S on pretreated mesh textile and an A260 of 1.043 ± 0.186 on pretreated CP55/45 against –0.007 ± 0.033 for wild-type. Esterase ranking did not predict textile performance.

**Discussion:**

Fold improvements over wild-type were preserved whether or not textiles were pretreated, with no hierarchy change detected across substrate formats, indicating that crystallinity is not the dominant variable governing relative variant performance. Physical features that persist regardless of crystallinity state are the more probable constraints on enzyme–substrate engagement. Wild-type gained little from crystallinity reduction alone. These results suggest that the capacity to engage a physically constrained polymer surface may be a productive target for engineering textile-active PET hydrolases.

## 1 Introduction

The rapid growth of PET-based textile production has created a major recycling problem. A central reason is that textile-derived PET is much harder to recycle than its bottled counterparts. PET fibers present unique recycling challenges because of their higher crystallinity and lower purity compared to bottle-grade PET, which hinders efficient separation and depolymerization (Enking et al., 2025). Garments further contain dyes, finishes, and blended fibers such as cotton, nylon, and elastane, which reduce feedstock uniformity and interfere with separation and recycling (Enking et al., 2025). In 2023, synthetic fiber production exceeded 124 million tons, with polyester/PET making up the largest share, highlighting its central role in global textile waste (Enking et al., 2025). Despite representing over half of all textile fibers, PET textiles remain largely overlooked in recycling efforts. In 2021, 99% of recycled polyester came from post-consumer bottles, while less than 1% was recovered from discarded garments (Textile Exchange, 2024). As a result, garments and household textiles have become a major and rapidly expanding end-of-life destination.

Cotton–PET blends are particularly challenging because the fibers are physically intertwined and additive-rich, making straightforward physical separation difficult and often shifting feasibility toward dissolution or selective degradation approaches (Wang and Salmon, 2022). Consequently, most discarded textiles are incinerated or landfilled, producing significant environmental impacts, including over 30 kg CO_2_ equivalents per kilogram of polyester fabric and widespread microfiber contamination across aquatic, terrestrial, and even Arctic ecosystems (Niinimäki et al., 2020; Palacios-Mateo et al., 2021). There is an urgent need for effective, sustainable recycling strategies capable of addressing the structural and compositional complexity of PET-based textiles while mitigating their environmental impact.

Industrial recycling of PET from cotton-blended textiles is conventionally achieved through mechanical or chemical processes, each presenting distinct advantages and inherent limitations. Mechanical recycling is a mature and scalable approach. However, it is primarily effective for high-purity, single-material PET textiles, which are rarely encountered in real-world markets. Dyes, finishes, and other additives contaminate the melt during repeated processing, degrading the quality of recycled PET and often resulting in downcycling (Enking et al., 2025). Chemical recycling can accommodate the compositional complexity of blended textiles, yet it imposes significant operational challenges. Cotton–polyester blends require extensive pretreatment, as the interlacing of PET with natural fibers, additives, and colorants restricts reagent accessibility, necessitating elevated temperatures, concentrated reagents, or energy-intensive thermo-chemical processes such as pyrolysis (>400 °C) and glycolysis (180 °C–240 °C), which generate oligomeric byproducts that are difficult to isolate (Lange, 2021; Enking et al., 2025). This creates tradeoffs across conventional routes. Mechanical and thermo-mechanical processing generally require high-purity input, while chemical and high-temperature routes can increase energy demands and separation burdens in mixed, additive-containing feedstocks (Lange, 2021; Enking et al., 2025). These limitations have motivated enzymatic PET depolymerization as a milder and more selective recycling strategy. However, its practical value depends on overcoming a central unresolved problem: efficient PET hydrolysis within complex, blended textile matrices.

The discovery of PET-degrading enzymes has enabled depolymerization of PET under such gentler and environmentally compatible conditions. PET-degrading enzymes catalyze hydrolysis of ester bonds along the PET backbone, releasing terephthalic acid (TPA) and soluble intermediates such as bis(2-hydroxyethyl) terephthalate (BHET) and mono (2-hydroxyethyl) terephthalate (MHET) (Kawai et al., 2020). Reaction efficiency, however, is strongly governed by polymer accessibility. A major barrier to enzymatic PET recycling is that hydrolysis depends not only on enzyme activity, but also on the physical accessibility and mobility of PET chains. Because PET is semi-crystalline, enzymes hydrolyze it more effectively when polymer chains have enough mobility to enter and interact productively with the active site, often near PET’s glass transition temperature (Tg, ∼70 °C) (Kawai et al., 2020). However, this requirement creates a difficult constraint for textile-relevant recycling: enzymes must remain active and structurally stable under prolonged high-temperature conditions, while also acting on fibers that are more crystalline, blended, and physically restricted than ideal model substrates. This thermal–stability tradeoff helps explain why enzymatic PET recycling studies often rely on simplified PET films or powders rather than complex textile materials.

Although protein engineering has improved PET hydrolase performance, enzymatic recycling remains disproportionately focused on idealized substrates rather than textile-grade materials, a critical limitation because crystallinity can strongly suppress enzymatic hydrolysis. Most studies have focused on virgin or low-crystallinity PET, while post-consumer textile fibers typically exhibit higher crystallinity and lower purity, limiting enzymatic accessibility and slowing hydrolysis (Enking et al., 2025; Thomsen et al., 2023b). Mixed cotton–PET textiles further complicate depolymerization, as the crystalline cellulose in cotton reduces substrate accessibility and prevents uniform enzyme activity (Boondaeng et al., 2023; El Darai et al., 2024; Kaabel et al., 2023). Although pretreatments such as melt-amorphization and alkaline treatment can increase PET accessibility by disrupting surface crystallinity and exposing hydrolysable ester bonds, their effectiveness is limited in heterogeneous textile waste, where dyes, finishes, cotton fibers, and uneven PET surface exposure create inconsistent enzyme–substrate contact (El Darai et al., 2024; Boondaeng et al., 2023). Together, these factors define an under-addressed gap. Enzymes optimized on simplified PET substrates may not retain sufficient activity or operational stability on fiber-based, cotton-blended textiles under near-Tg conditions (Thomsen et al., 2022).

Among thermostable cutinases, *Thermobifida fusca* cutinase 2 (*Tf*Cut2) is a particularly strong scaffold for enzymatic textile recycling. It exhibits improved thermal stability relative to other bacterial cutinases (Carr et al., 2020), enabling PET hydrolysis under moderately high temperatures, and shares significant sequence similarity with both Leaf-branch compost cutinase (LCC) and PETase, including a conserved GXSXG motif, a Ser–His–Asp catalytic triad, and an α/β-hydrolase fold (Buchholz et al., 2022). Structure-guided and computational studies have identified residues involved in PET binding and catalysis, supporting rational variant design (Mrigwani et al., 2023), while stability engineering has shown that thermostability and calcium independence can be improved through engineered disulfide bridges (Then et al., 2016). However, these advances do not resolve the central textile-recycling problem: whether engineered PET hydrolases can remain effective on complex cotton–PET substrates. Therefore, this study uses *Tf*Cut2 as a platform to develop and test variants directly against cotton-blended textiles, linking enzyme engineering to the substrate complexity that limits practical textile recycling. We compare wild-type *Tf*Cut2 and engineered variants across model PET substrates, PET textile fibers, and cotton–PET blends to determine how enzyme performance changes with substrate complexity. Because fiber-based textiles present restricted and heterogeneous PET surface exposure, we incorporate alkaline pretreatment as an accessibility-enhancing step before enzymatic depolymerization. This study establishes a cross-substrate evaluation framework for PET-degrading *Tf*Cut2 variants and supports the development of pretreatment-assisted enzymatic recycling strategies for circular processing of blended textile waste.

## 2 Methods and materials

### 2.1 Conserved residue identification

The amino-acid sequence of *Tf*Cut2, derived from Protein Data Bank entry 5ZOA (PDB ID: 5ZOA; Berman et al., 2000; Dong et al., 2020), was queried against the NCBI ClusteredNR database using BLASTP (Altschul et al., 1990; 1997; Johnson et al., 2008; Sayers et al., 2025). To expand conservation analysis beyond Thermobifida homologs, *Tf*Cut2 was aligned with 13 plastic-degrading enzymes (*Ideonella sakaiensis, T. fusca, Humicola insolens, Thermobifida cellulosilytica, Thermobifida alba, Fusarium solani pisi, Saccharomonospora viridis, Fusarium oxysporum, Aspergillus fumigatus, Triticum aestivum, Thermomyces lanuginosus, Thermomonospora curvata, Pseudomonas aestusnigri*) (Maurya et al., 2020). Multiple sequence alignment (MSA) was performed using Clustal Omega (Sievers et al., 2011). Residues previously reported as conserved in the literature were excluded from the candidate mutation set (Joo et al., 2018; Furukawa et al., 2019; Kawai et al., 2020).

### 2.2 Molecular docking simulations

Three-dimensional structures of *Tf*Cut2 and variants were predicted using the AlphaFold three web server using full-length amino-acid sequences (Abramson et al., 2024). *Tf*Cut2 5ZOA was selected as the structural reference rather than the engineered variant *Tf*Cut2 KW3, as AlphaFold three predictions for KW3 contained extended low-confidence regions that could significantly affect docking simulations. For each sequence, the top-ranked AlphaFold three model was used for downstream docking. Receptor preparation was performed using UCSF Chimera DockPrep (Pettersen et al., 2004). Docking was then carried out using AutoDock Vina v1.1.2 (Trott and Olson, 2010). The docking grid box was centered at x = 0, y = 0, and z = 0, with dimensions of 50 Å × 50 Å × 50 Å. The exhaustiveness parameter was set to 8, the number of binding modes was set to 10, and the energy range was set to 3 kcal/mol. The predicted binding poses were ranked according to the AutoDock Vina binding affinity score, and the top-ranked pose was used for downstream analysis.

### 2.3 In silico mutagenesis

Alanine scanning was performed by mutating each unconserved position to alanine, followed by docking as described above (Cunningham and Wells, 1989). Eight positions were then selected for site-saturation mutagenesis based on their proximity to the active site and their significant impact on binding affinity upon alanine substitution (65, 82, 173, 180, 183, 188, 193, 231). An additional eight positions were selected for their minimal contribution to active-site interactions and low impact on binding affinity (63, 77, 83, 184, 196, 197, 250, 251). Site-saturation mutagenesis was then performed at these 16 selected positions, substituting all 20 canonical amino acids and ranking variants by ΔΔGbind (Reetz and Carballeira, 2007).

MutCompute, a three-dimensional convolutional neural network (3D-CNN) available as a deep learning server (Shroff et al., 2020), was applied to the X-ray crystal structure of wild-type *Tf*Cut2 (PDB ID: 5ZOA, resolution: 1.54 Å). For each position, MutCompute predicts the probability of every amino acid given its structural environment. A total of 43 positions were identified where the wild-type residue was not the highest-probability choice. MutCompute outputs an avg_log_ratio, representing the predicted probability of the suggested residue relative to the wild-type residue, and the top mutations from the conserved and unconserved regions were chosen as single mutants. Conserved residues were excluded from the 43 positions, and the remaining substitutions were incorporated into the MC-nc27 variant (MutCompute non-conserved, 27 substitutions, Full MutCompute suggestion).

### 2.4 Plasmid construction and molecular cloning

The gene encoding *Tf*Cut2 from *T. fusca* (PDB: 5ZOA) was synthesized as codon-optimized DNA fragments for *Escherichia coli* expression (Integrated DNA Technologies). The pET-28a vector was used as the expression backbone. The vector was linearized by digestion with EcoRI-HF and XhoI (New England Biolabs). Recombinant plasmids were constructed using the NEBuilder HiFi DNA Assembly Kit following the manufacturer’s instructions. *Tf*Cut2-5ZOA and G62A/P193H/S197D, H77R/D204C/E253C, and A65H/L90A/I213S were constructed using forward primer 5′-TGG​GTC​GCG​GAT​CCG​AAT​TCG​CGA​ACC​CCT​ATG​AAC​GCG-3′ and reverse primer 5′-TGG​TGG​TGG​TGG​TGC​TCG​AGG​AAC​GGG​CAT​GTT​GAG​CGA-3’. D12S, T234L, and MC-nc27 (27-substitution MutCompute construct) were constructed using forward primer 5′-TGG​GTC​GCG​GAT​CCG​AAT​TCG​CGA​ACC​CGT​ATG​AAC​GCG-3’. D12 S and T234L were constructed using reverse primer 5′-TGG​TGG​TGG​TGG​TGC​TCG​AGA​AAC​GGG​CAG​GTG​CTG-3’. MC-nc27 (MutCompute non-conserved, 27 substitutions, full MutCompute suggestion) was constructed using reverse primer 5′-TGG​TGG​TGG​TGG​TGC​TCG​AGA​AAC​GGC​GCG​GTG​C-3’. Assembly products were transformed into *E. coli* DH5α (Yeastern Biotech) for plasmid propagation. All final constructs were sent for DNA sequencing to Genomics BioSci and Tech to confirm the presence of intended mutations and the absence of undesired errors.

### 2.5 Protein expression and purification

Plasmids were transformed into *E. coli* BL21 (DE3) (Real Biotech Corporation). Liquid culture in 5 mL TB medium was added to 250 mL TB medium with 25 μg/mL Kanamycin and incubated at 37 °C until OD600 reached 0.5–1.0. IPTG was then added to a final concentration of 1 mM, and cultures were incubated at 16 °C for 20 h. Cells were harvested by centrifugation, and the pellet was resuspended in 10 mL CelLytic B lysis buffer (Sigma-Aldrich). For larger-scale extraction, we added lysozyme (0.2 mg/mL) (Real Biotech Corporation) to break the cell wall. DNase I (25 μg/mL) was included to reduce viscosity. A protease inhibitor cocktail (EDTA-Free, 100× in DMSO, TargetMol) was also added to prevent degradation. The mixture was shaken at room temperature for around 15 min. After lysis, the insoluble protein and debris were removed by centrifugation. The soluble lysate was purified by a HisPur Ni-NTA spin column (Thermofisher), spun at 1,251 rpm for 2 min. *Tf*Cut2 and its variants were then eluted using 150 mM imidazole, and the flow-throughs were resolved by SDS-PAGE. They were then washed 3 times with 20 mM imidazole, and analyzed by Western blot using His-Tag antibody (AD1.1.10, Santa Cruz Biotechnology) to verify the protein expression, and Coomassie blue staining to verify the purity of flow-through. The flow-through was dialyzed against 50 mM sodium borate (pH 8.0) on ice using Slide-A-Lyzer dialysis cassettes (10K MWCO, Thermofisher).

After dialysis, the protein concentration was measured by the Bradford assay (BioRad). Enzyme kinetic analysis was used to standardize the active concentration of *Tf*Cut2 across purification batches. Activity was measured via p-nitrophenyl butyrate (pNPB) assay by recording UV absorbance at 405 nm every minute. The initial reaction rate was determined by calculating the linear slope of the absorbance versus time graph, specifically selecting the latest interval where the correlation coefficient (R^2^) exceeded 0.99. Since concentration of substrate is large, the initial rate of reaction can approximate the maximum reaction rate, which is directly proportional to the *Tf*Cut2 enzyme concentrations.

Relative functional enzyme concentrations were calculated by dividing the slope of the new batch by the slope of a reference batch. The reciprocal of this ratio provided a correlation factor used to adjust the enzyme stock volume. This standardized the input, ensuring a constant active *Tf*Cut2 concentration of 27.8 μg/mL across all subsequent assays.

### 2.6 Esterase activity assay

The esterase activity of *Tf*Cut2 and its variants was analyzed by pNPB assay. To determine the optimal reaction conditions for *Tf*Cut2 and its variants, the reactions were performed under the following conditions: (1) 200 mM Tris-HCl across a pH range of 6.0–8.5; (2) 200 mM Tris-HCl (pH 7.0–8.5) supplemented with 10 mM CaCl_2_; and (3) 100 mM HEPES (pH 7.0–8.5) supplemented with 10 mM CaCl_2_. The final concentration of enzymes was 5 μg/mL and substrate 4-nitrophenyl butyrate (pNPB) was 2 mM. The reactions were incubated at 35 °C–70 °C for 30 min. The absorbance was measured by a microplate reader (Clubio FlexA-200) at 405 nm. Reactions were performed in technical triplicate within each independent batch. Wild-type *Tf*Cut2 was assayed across five independent batches at 35 °C–55 °C and two independent batches at 60 °C–70 °C; engineered variants G62A/P193H/S197D, H77R/D204C/E253C, and A65H/L90A/I213 S were assayed across three independent batches at 35 °C–55 °C and one batch at 60 °C–70 °C; and D12S, T234L, and MC-nc27 were assayed across two independent batches at 35 °C–55 °C and one batch at 60 °C–70 °C.

### 2.7 Optimized condition for PET degradation

To optimize PET degradation conditions, high-throughput screening was conducted using small PET film strips (0.5 cm × 0.25 cm) incubated with *Tf*Cut2 and its variants in 8-strip PCR tubes. An enzyme concentration of 5 μg/mL was used for this screening step to maintain consistency with the pNPB esterase assay and allow direct comparison between the two assay formats. These were incubated with the enzyme in 100 mM and 500 mM HEPES buffer (pH 7–8.5) with 10 mM CaCl_2_. They were incubated with temperatures (35 °C–70 °C) shaking for 48 h. Afterward, the supernatant was transferred to a 96-well UV microplate F (Basic Life Bioscience), and absorbance at 260 nm was measured using a microplate reader (Clubio FlexA-200). As verified by Zhong-Johnson et al. (2021), A260 was used as a relative indicator of the combined release of the aromatic PET-degradation products TPA, MHET, and BHET. Because these products were not individually resolved and have different absorbance contributions, the measurements were used only for comparisons between variants and conditions, rather than as an absolute quantification of PET depolymerization. Screening in both buffer conditions was performed across three independent batches (n = 3) per variant × temperature × pH combination. At this enzyme concentration, degradation activity against textile substrates was negligible. Enzyme loading was therefore increased for subsequent PET film and textile degradation assays (see below).

### 2.8 PET film degradation

To assess the enzymatic PET degradation efficiency of wild-type *Tf*Cut2 and its variants, we scaled up the PET degradation using 27.8 μg/mL enzyme (equivalent to 1 μM, based on the *Tf*Cut2 mature protein molecular weight of ∼27.8 kDa), consistent with the enzyme loading used in prior *Tf*Cut2 PET-degradation studies (Furukawa et al., 2019), in 500 mM HEPES (pH 7.5) with 10 mM CaCl_2_, and eight pieces of PET film (0.5 cm × 3 cm) in a 2 mL microtube in one reaction. After 51.8 °C at 800 rpm incubation on thermalshaker (DB100-C, JoanLab) for 48 h, supernatant was collected for the further analysis. The PET films were washed with 1% SDS and 95% ethanol, then rinsed with deionized water and dried overnight. The weight of dried PET films was measured by analytic balance (MG214Ai, BEL Engineering). Reactions were performed in three independent batch replicates (n = 3) per variant.

### 2.9 Textile alkaline pretreatment

To determine suitable pretreatment conditions for enzymatic degradation, textile samples were subjected to alkaline–thermal pretreatment across a range of durations and submitted to COMPEQ Manufacturing Co., Ltd. for crystallinity quantification by differential scanning calorimetry (DSC). The submitted samples comprised pure PET fiber (untreated, and pretreated for 4 and 8 h), pure PET knit textile (untreated, and pretreated for 4, 8, and 20 h), and pure PET mesh textile (untreated, and pretreated for 4 and 8 h), all treated with 15% (w/v) NaOH at 80 °C. Because samples for DSC were taken directly after pretreatment, prior to the wash and dry step, crystallinity could be determined for all treatment durations, including the 8 h and 20 h conditions whose textiles did not survive rinsing intact. Longer treatments produced greater crystallinity reduction but compromised structural integrity: the 20 h treatment disintegrated the textile into fine powder, and the 8 h treatment left it too fragile to withstand rinsing. Treatment with 15% NaOH at 80 °C for 4 h reduced crystallinity while preserving the textile structure, and was therefore selected as the pretreatment condition for subsequent enzymatic degradation assays.

Using the selected condition, textile samples (PET or PET–cotton blends) were cut into small pieces (approximately 0.1 × 0.5 cm; total weight around 2 g) and immersed in 20 mL of 15% (w/v) NaOH in 50 mL centrifuge tubes, then incubated in a dry bath at 80 °C for 4 h. Following pretreatment, textiles were thoroughly washed under running tap water for 5 min, rinsed with deionized water, and dried at 55 °C for 48 h.

### 2.10 PET textile degradation

To validate the degradation efficacy of the engineered enzymes on commercial textiles, PET textile fibers, pure PET textiles, and PET/cotton blended textiles were purchased from retail sources. The PET textile fibers were obtained from Toung Loong Textile MFG. For the pure PET fabrics, PET textile A (knit) was purchased from a local market (Dadaocheng, Taipei City), while PET textile B was a Kiprun Run 100 garment (mesh, Decathlon, France). Regarding the blended fabrics, 55% cotton/45% PET (mesh) and 65% cotton/35% PET (mesh) blends were acquired from NET (Taiwan). Blend compositions (55% cotton/45% PET and 65% cotton/35% PET) are as specified by the manufacturer and were not independently verified.

All textiles were cut into 0.1 × 0.5 cm pieces. Subsequently, 3 g of the prepared textiles were subjected to alkaline pretreatment, as previously described. Following pretreatment, enzymatic degradation was performed using 50 mg of the treated textiles or a sample mass containing an equivalent amount of PET fibers. Reaction conditions were identical to those described in the PET Film Degradation section. For pure PET textile substrates (fiber, knit, and mesh), assays were performed with five independent batch replicates (n = 5) for pretreated samples and three independent batch replicates (n = 3) for untreated samples. For cotton–PET blended textiles (CP65/35 and CP55/45), assays were performed with three independent batch replicates (n = 3) per condition (pretreated and untreated).

### 2.11 Statistical analysis

Analyses were performed in R (version 4.5.0, R Core Team, 2024). Absorbance values were blank-corrected per replicate before averaging. Each n reflects an independent biological replicate (separate batch).

Variant-vs-wild-type comparisons used Dunnett’s test (Dunnett, 1955) via the glht () function in the multcomp package (version 1.4–26, Hothorn et al., 2008), applied to a linear model of activity by enzyme, fit separately per substrate/pretreatment condition.

To test whether pretreatment shifted the performance hierarchy, wild-type and MC-nc27 were excluded, values were normalized within each pure PET fiber type/treatment to the mean of remaining variants, and a linear model (normalized activity ∼ treatment × enzyme) was fit per pure PET fiber type; the interaction term tested for hierarchy change. This test was applied only to the pure PET formats, for which crystallinity was measured before and after pretreatment.

Optimal temperature/pH per variant were estimated by response surface methodology. Temperature and pH were centered/scaled and fit via lm () as a second-order polynomial (linear, quadratic, and interaction terms), separately for each variant. Outliers were flagged per Variant × Temperature × pH group using a modified Z-score (MAD-based, threshold = 3.5) and excluded. Per-variant optima were taken as the maximum predicted value over an 80 × 80 grid; model fit was assessed via R^2^ and RMSE. The pooled global optimum (51.8 °C, pH 7.5) was obtained using an analogous RSM procedure applied to absorbance values averaged across active variants (excluding the final variant MC-nc27).

Predicted activity at pH 8.5 was evaluated using each variant’s fitted RSM model at that variant’s optimal temperature and expressed as a fraction of the variant’s predicted peak activity within the tested pH range. Both quantities were evaluated at each variant’s point-estimate optimal pH, and the standard error of the resulting ratio was obtained by the delta method, using the fitted model’s coefficient covariance matrix. MC-nc27 was excluded due to negligible, noise-dominated activity across all conditions.

## 3 Results

### 3.1 Design of TfCut2 mutant variants

To optimize *Tf*Cut2 for improved efficiency and stability, the crystal structure of *Tf*Cut2 (PDB: 5ZOA) was used *in silico* alanine scanning mutagenesis. Sixteen residues were identified and subjected to *in silico* site-saturation mutagenesis (SSM). At each position, the 19 possible amino-acid substitutions were generated and scored for ΔΔG of PET binding. Of the 304 substitution–energy pairs most were neutral or less thermodynamically favorable. A smaller subset returned negative ΔΔG values, indicating predicted improvement in PET-binding affinity (Table 1). Negative-ΔΔG mutations were then combined with MutCompute predictions to form the candidate pool for variant design.

**TABLE 1:** Summary of TfCut2 variants and their design rationale. WT = wild-type TfCut2 (PDB: 5ZOA).

Mutations	Design strategy	Primary target
G62A/P193H/S197D	Designed second (non-native) triad and MHET inhibition	Catalytic efficiency and MHET inhibition
H77R/D204C/E253C	High-affinity binding and disulfide bridge	PET binding affinity and thermostability
A65H/L90A/I213 S	LCC-derived substrate-cleft widening and new docking position	PET accessibility and MHET inhibition relief
D12 S	MutCompute (non-conserved, highest avg_log_ratio)	Structural stability
T234L	MutCompute (conserved, top global rank)	Thermostability
MC-nc27	MutCompute non-conserved, 27 substitutions (full suggestion)	Thermostability accumulation

Finally, six variants were designed, each targeting a distinct mechanistic hypothesis. (Table 1). G62A/P193H/S197D combines G62A, a substitution in the PET-binding groove reported to relieve MHET product inhibition (Wei et al., 2016), with P193H and S197D, two substitutions selected to complete a second, non-native Ser-His-Asp-type triad together with the native residue Ser194, testing whether an independent catalytic site elsewhere on the protein could supplement the native triad’s activity. H77R/D204C/E253C pairs the high PET binding affinity substitution H77R with the D204C/E253C disulfide bridge, previously shown to raise the melting temperature and reduce Ca^2+^ dependence in *Tf*Cut2 (Then et al., 2016). A65H/L90A/I213 S was informed by the Leaf-branch compost cutinase (LCC) template (Dong et al., 2020), and A65H was also chosen for its high binding affinity to PET. D12S, T234L, and MC-nc27 were derived from MutCompute predictions. The D12S substitution exhibited the largest avg_log_ratio, suggesting the greatest predicted stability of a residue compared to the wild type, the native amino acid among non-conserved positions. The T234L mutation was the top-ranked single substitution overall, located at a conserved position where substitutions would be expected to interfere with catalytic performance. MC-nc27 aggregated all non-conserved MutCompute-predicted stabilizing mutations and was designed to probe cumulative thermostabilization. These six variants were then evaluated for enzyme activity and thermal stability.

### 3.2 Optimal activity condition for different variants

To evaluate the optimal reaction condition of the proposed enzyme, a para-nitrophenyl butyrate (pNPB) assay was performed, in which pNPB is hydrolyzed by the *Tf*Cut2 enzyme to assess its esterase activity. A HEPES buffer supplemented with calcium was selected for subsequent experiments because previous studies demonstrated that Ca^2+^ can significantly enhance the thermal stability of *Tf*Cut2 by increasing its melting temperature (Then et al., 2016). Six different temperatures (35, 45, 55, 60, 65, and 70 °C) and four different pH conditions (pH 7.0, 7.5, 8.0, and 8.5) were screened to determine the optimal conditions for *Tf*Cut2 enzymatic activity and stability. Response surface methodology (RSM) predicted the highest pNPB activity at the lowest temperature tested (35 °C) for most variants, rather than at a defined interior optimum. A65H/L90A/I213S was the exception, showing an interior local maximum at approximately 38 °C (Table 2; Supplementary Figure S1). All variants except MC-nc27 showed a comparable optimal pH range of 7.0–7.5 (Table 2) and comparable activity under their respective optimal conditions. MC-nc27 yielded near-zero absorbance across all tested temperatures and pH values (Supplementary Figure S1). However, degradation results from pNPB cannot be extrapolated for hydrophobic and insoluble PET (Pirillo et al., 2021).

**TABLE 2:** Optimal temperature and pH per enzyme variant, comparing the pNPB activity assay with the PET film high-throughput screening (HTS) condition.

Enzyme	pNPB assay		PET film (HTS)	
	Temp (°C)	pH	Temp (°C)	pH
Wild-type	35.0	7.3	51.8	7.6
G62A/P193H/S197D	35.0	7.5	50.9	7.7
H77R/D204C/E253C	35.0	7.3	51.8	7.0
A65H/L90A/I213 S	38.1	7.0	50.9	7.8
D12 S	35.0	7.2	52.7	7.5
T234L	35.0	7.2	53.2	7.6
MC-nc27	35.0	7.8	35.0	8.5

**FIGURE 1:**
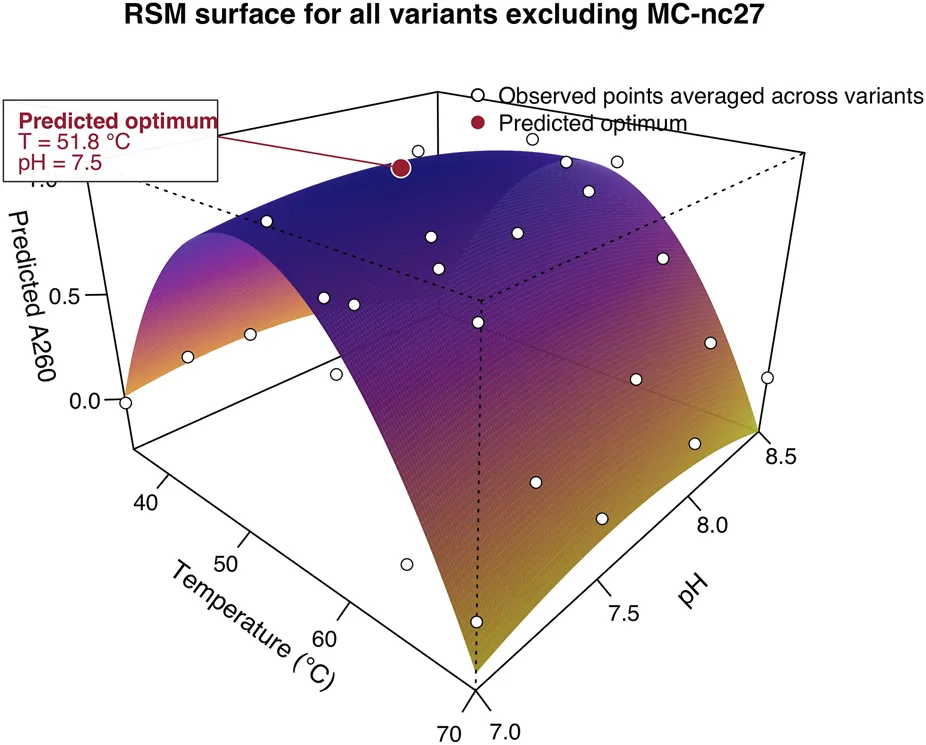
Overall response surface methodology (RSM) model of PET film degradation activity across four pH conditions (pH 7.0, 7.5, 8.0, and 8.5) and seven temperatures (35, 45, 50, 55, 60, 65, and 70 °C). The surface was generated using averaged A_260_ values from wild-type TfCut2 and the five active engineered variants, while MC-nc27 was excluded due to negligible activity. White circles represent experimental observations used for model fitting. The global maximum was identified at approximately 51.8 °C and pH 7.5.

Therefore, PET film screening was then performed across four pH conditions (pH 7.0, 7.5, 8.0, and 8.5) and seven temperatures (35, 45, 50, 55, 60, 65, and 70 °C) in HEPES/CaCl2 buffer. Degradation efficiency was quantified by measuring UV absorbance at 260 nm (A260), corresponding to the cumulative release of terephthalic acid (TPA), mono (2-hydroxyethyl) terephthalate (MHET), and bis(2-hydroxyethyl) terephthalate (BHET). RSM identified an optimal condition of approximately 51 °C–53 °C and pH 7.0–7.8 for all active variants (Supplementary Figure S2; Table 2). The data revealed similar optimal temperatures among all variants and comparable optimal pH conditions for most variants, except for H77R/D204C/E253C, whose optimal pH (7.0) was modestly lower than the other active variants (7.5–7.8) (Table 2). Moreover, markedly different optimal conditions were observed in PET film degradation assays compared with the pNPB assay, particularly with respect to temperature. To determine a unified reaction condition for further experimentation, the absorbance values obtained from PET film degradation assays of the active variants and wild-type (MC-nc27 is excluded due to its lack of detectable activity) were averaged and integrated into a single response surface model. Based on this combined analysis, the global optimal condition was determined to be 51.8 °C at pH 7.5 (Figure 1). This unified condition was selected to standardize subsequent cross-variant comparisons.

### 3.3 PET film degradation activity

**FIGURE 2:**
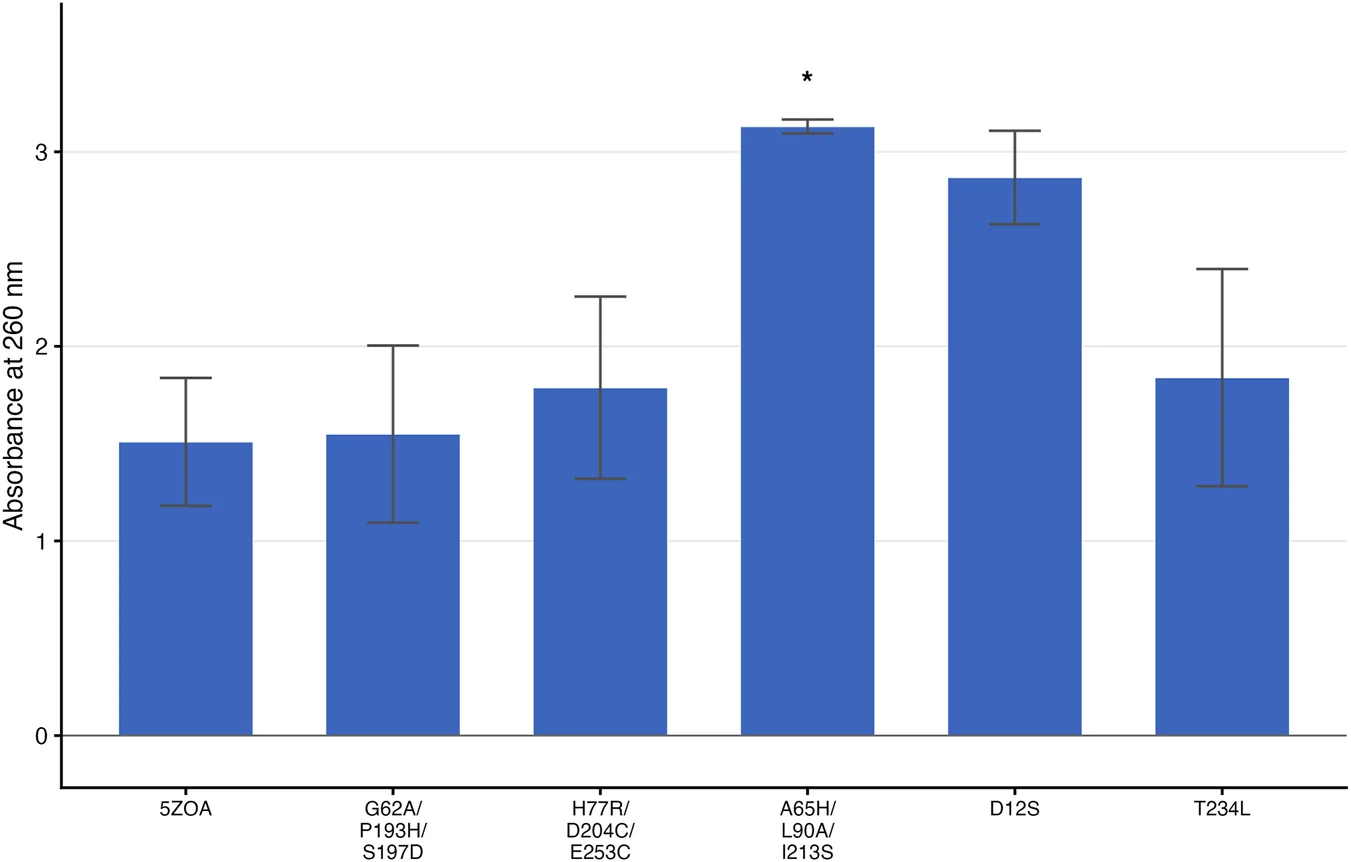
PET film degradation by TfCut2 wild-type and five active engineered variants, quantified by A_260_. Conditions: 51.8 °C, pH 7.5, 500 mM HEPES/CaCl_2_. Bars represent means of three independent batch replicates (n = 3); error bars represent 1xSEM. Statistical analyses using the Dunnett test were performed to compare WT and variant enzymes. Significance levels are indicated as follows: #P < 0.1, *P < 0.05, **P < 0.01, ***P < 0.001.

The results showed that A65H/L90A/I213 S exhibited the highest mean A260 (3.13 ± 0.04) (Figure 2), corresponding to a 2.1-fold increase over WT (1.51 ± 0.37). D12 S displayed the second highest mean (2.87 ± 0.22), although it exhibited greater variability among replicates, followed by T234L (1.84 ± 0.58), H77R/D204C/E253C (1.79 ± 0.47), and G62A/P193H/S197D (1.55 ± 0.45). MC-nc27 showed no detectable activity against PET film and was excluded from subsequent degradation comparisons and statistical analyses on textile substrates. Full results for MC-nc27 across all substrates are reported separately below. To determine whether the degradation trends observed among the five active variants and wild-type were maintained across more complex polymeric materials, these six constructs were evaluated using textile-based substrates.

### 3.4 Assessment of textile degradation capability

#### 3.4.1 Alkaline pretreatment

Differential scanning calorimetry (DSC) confirmed that alkaline pretreatment reduced the crystallinity (Xc) of the PET textile substrates (Table 3), consistent with prior reports that alkaline pretreatment enhances the efficiency of enzymatic PET degradation (Thomsen et al., 2022). Untreated knitted and mesh textiles had relatively high crystallinity, at 47.9% and 44.1%, respectively, and crystallinity decreased with increasing treatment duration. The selected condition, 15% NaOH at 80 °C for 4 h, lowered crystallinity to 22.1% in the knitted textile and 22.7% in the mesh textile while preserving the overall textile structure (see Methods). Longer treatments reduced crystallinity further but compromised structural integrity, precluding their use in enzymatic degradation assays.

**TABLE 3:** Crystallinity index (Xc, %) of PET substrates measured after alkaline (NaOH) pretreatment at 80 °C for varying durations.

Treatment	Substrate	Xc (%)
Untreated	Pure PET fiber	36.5
15% NaOH, 80 °C, 4 h	Pure PET fiber	11.7
15% NaOH, 80 °C, 8 h	Pure PET fiber	5.6
Untreated	Pure PET knit textile	47.9
15% NaOH, 80 °C, 4 h	Pure PET knit textile	22.1
15% NaOH, 80 °C, 8 h	Pure PET knit textile	16.7
15% NaOH, 80 °C, 20 h	Pure PET knit textile	1.5
Untreated	Pure PET mesh textile	44.1
15% NaOH, 80 °C, 4 h	Pure PET mesh textile	22.7
15% NaOH, 80 °C, 8 h	Pure PET mesh textile	11.5

Alkaline-pretreated textiles exhibited greater *Tf*Cut2-mediated degradation compared with untreated controls. Across matched mutant–pretreatment combinations, pretreated PET fiber substrates exhibited an average 6.32-fold higher absorbance than untreated pure PET fiber textile substrates, while cotton–PET blended textiles showed an average 6.95-fold increase relative to untreated cotton–PET blended textiles (excluding zero values). These findings support the conclusion that substrates with lower crystallinity and greater surface accessibility are more susceptible to enzymatic degradation.

#### 3.4.2 Pure PET textile degradation

Different forms of pure PET textiles, including PET fiber, PET knit textile, and PET mesh textile, were first evaluated (Supplementary Figure S3). Wild-type *Tf*Cut2 exhibited consistently negligible degradation activity across nearly all pretreatment textile substrates. In contrast, G62A/P193H/S197D, H77R/D204C/E253C, A65H/L90A/I213S, D12S, and T234L all demonstrated measurable PET degradation activity, with A65H/L90A/I213S generally exhibiting the strongest and most consistent performance across the three PET substrate systems.

Pure PET mesh textile was the substrate on which variant improvement was most consistently captured, with all five engineered variants showing statistically significant increases in degradation activity relative to wild-type *Tf*Cut2, a pattern not uniformly observed across the other pure PET substrates. Although degradation activities among the active variants were generally comparable, A65H/L90A/I213S achieved higher degradation levels than other variants. A65H/L90A/I213S reached an A_260_ value of approximately 1.39 ± 0.17, corresponding to nearly a 14-fold increase relative to the wild-type *Tf*Cut2 baseline of approximately 0.097 ± 0.013.

**FIGURE 3:**
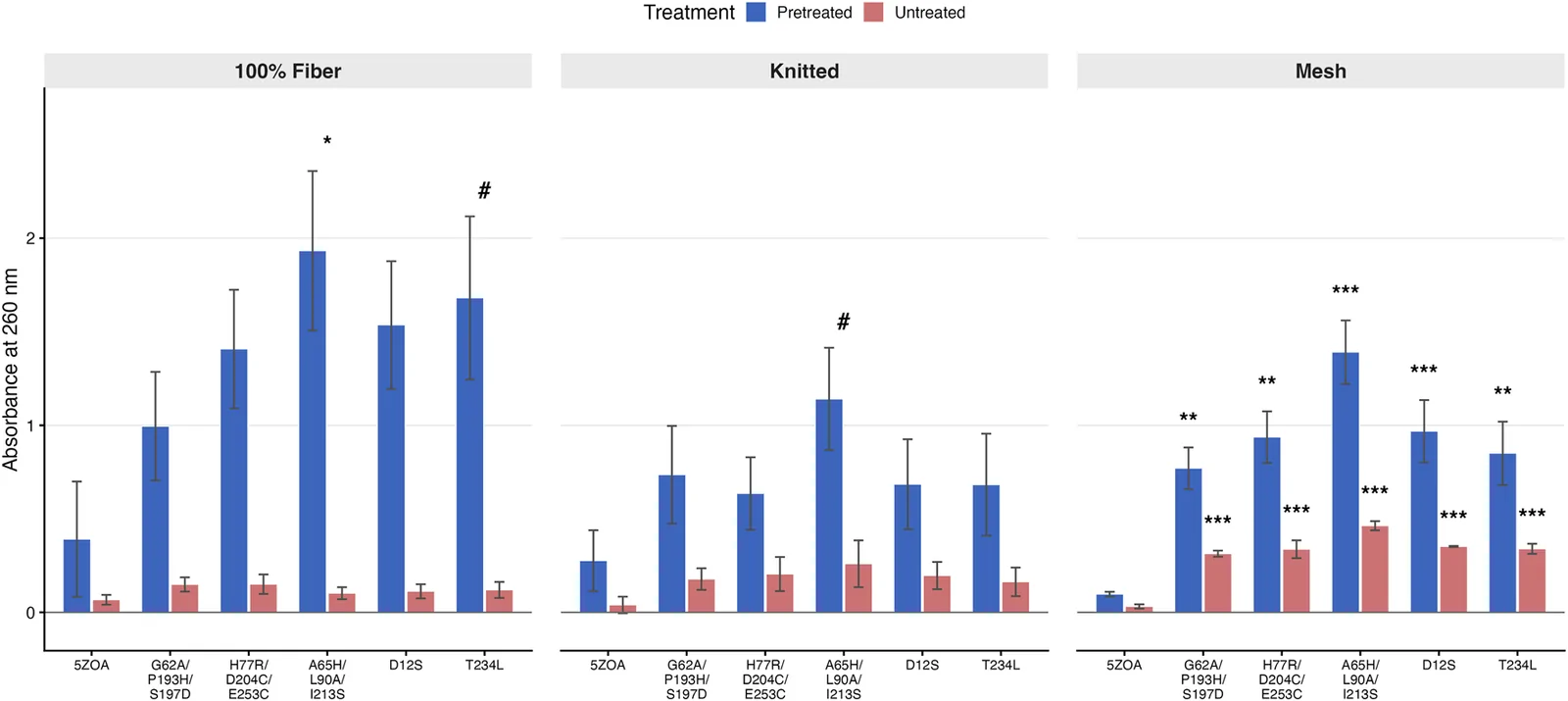
Enzyme activity of wild-type TfCut2 (5ZOA) and engineered variants across three pure PET textile substrates under untreated and NaOH-pretreated conditions. Activity was measured as absorbance at 260 nm (A260); error bars represent SEM. Data for pretreated textile is from five independent experimental trials (n = 5), while untreated textile is from three independent experimental trials (n = 3). Statistical significance relative to wild-type TfCut2 (5ZOA) was determined using Dunnett’s multiple comparison test. Significance levels are indicated as follows: #P < 0.1, *P < 0.05, **P < 0.01, and ***P < 0.001.

To select the most appropriate textile type for later experiments, both degradation performance and industrial relevance were considered. Although pure PET fiber substrate yielded the highest degradation results, it does not represent a form of textile waste encountered in industrial settings. Between the two textile forms, the engineered variants showed clearer differentiation from wild-type on pure PET mesh than on pure PET knit textile. Additionally, all active variants demonstrated relatively high degradation results on untreated mesh textile. A65H/L90A/I213S displayed a significant performance advantage on the mesh substrate compared to other enzyme variants (Figure 3).

#### 3.4.3 Cotton-PET blend textile degradation

Due to the clearer variant differentiation observed on pure PET mesh textile and its greater relevance to textile waste, two cotton–PET mesh blends, CP65/35 (65% cotton/35% PET) and CP55/45 (55% cotton/45% PET), were selected to evaluate the degradation performance of engineered *Tf*Cut2 variants on cotton–PET blended textiles. The blended textiles were subjected to the same alkaline pretreatment conditions as those used for pure PET textiles.

**FIGURE 4:**
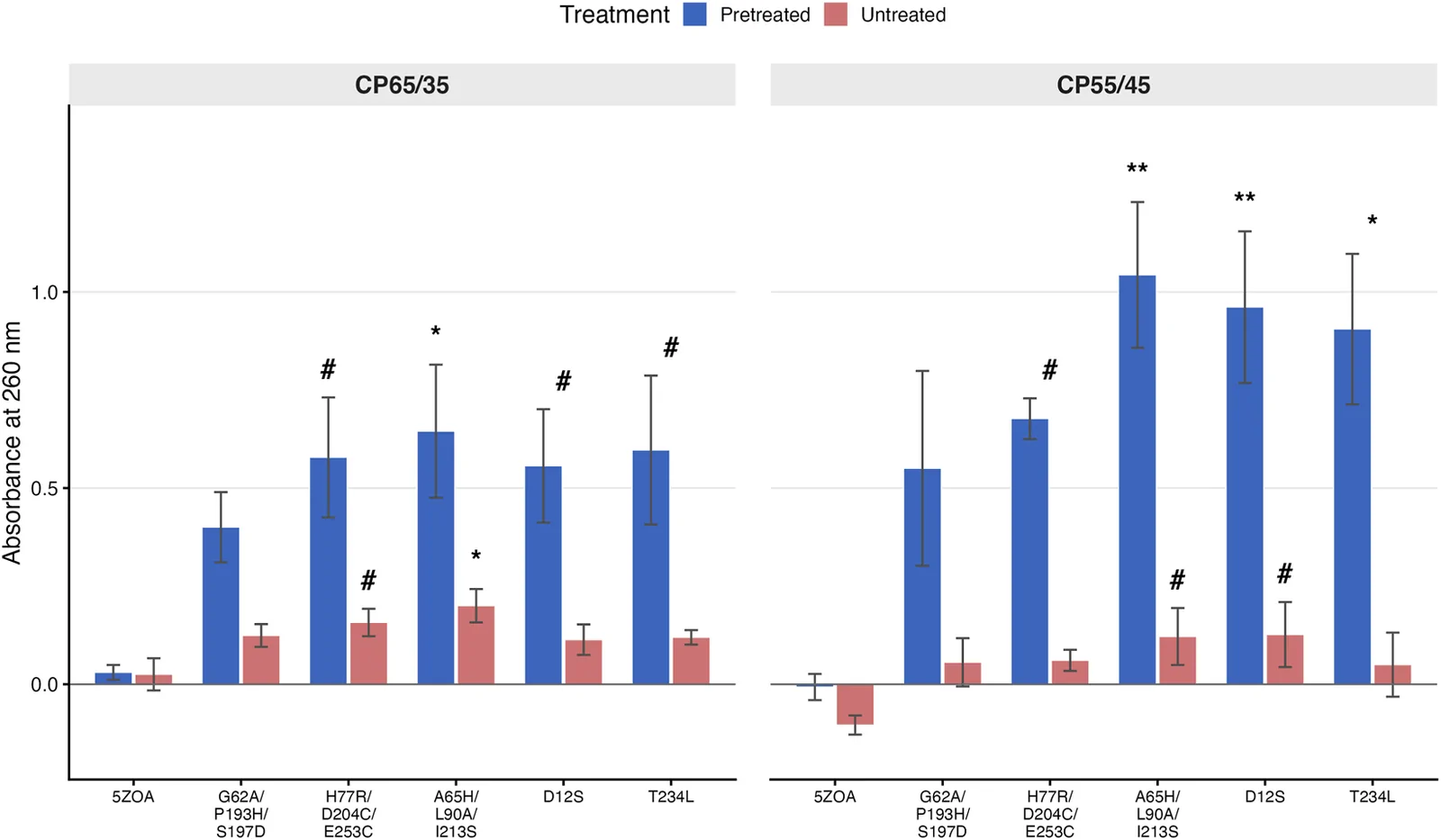
Enzyme activity of wild-type TfCut2 (5ZOA) and engineered variants across blended cotton–PET textile substrates CP65/35 and CP55/45 under untreated and NaOH-pretreated conditions. Activity was measured as absorbance at 260 nm (A260); error bars represent SEM from three independent experimental trials (n = 3). Statistical significance relative to wild-type TfCut2 (5ZOA) was determined using Dunnett’s multiple comparison test. Significance levels are indicated as follows: #P < 0.1, *P < 0.05, **P < 0.01, and ***P < 0.001.

Wild-type *Tf*Cut2 exhibited negligible degradation activity across all blend compositions and pretreatment conditions (Figure 4). In contrast, the engineered variants showed measurable but variable activity across the two cotton–PET blends. A65H/L90A/I213S and D12S consistently produced the highest degradation signals across substrate systems, while T234L showed an improvement in blended textiles relative to its relatively lower performance on pure PET film. H77R/D204C/E253C exhibited moderate yet significant improvements over wild-type *Tf*Cut2, whereas G62A/P193H/S197D displayed only modest, non-significant improvements.

Fiber composition had a pronounced effect on degradation performance. Across all active variants, the CP55/45 blend produced an average 1.7-fold higher absorbance than CP65/35, consistent with its lower cotton content. All active variants displayed broadly similar activities in the lower-PET CP65/35 blend, while the CP55/45 substrate generated greater differentiation between variants, with A65H/L90A/I213S, D12S, and T234L consistently outperforming G62A/P193H/S197D and H77R/D204C/E253C (Figure 4). A65H/L90A/I213 S showed the strongest PET-dependent performance trend, with its advantage over other variants becoming more pronounced as PET concentration increased from CP65/35 to CP55/45 and pure PET mesh textile. Under alkaline-pretreated CP55/45 conditions, A65H/L90A/I213S showed the highest mean absorbance of 1.043 ± 0.186; D12S and T234L showed similarly strong responses (0.961 ± 0.193 and 0.905 ± 0.192, respectively), corresponding to A_260_ increases of ∼1.050, ∼0.968, and ∼0.912 above the near-zero wild-type baseline (−0.007 ± 0.033).

#### 3.4.4 MC-nc27 activity across substrates

MC-nc27 was excluded from the primary degradation comparisons above due to negligible activity on PET film; its results across all remaining substrates are summarized here. MC-nc27 showed near-zero pNPB esterase activity across all tested temperature and pH combinations, in contrast to WT and the five other variants, which displayed comparable activity under their respective optimal conditions (Supplementary Figure S1). On PET film, MC-nc27 returned a blank-corrected A_260_ of −0.10 ± 0.07, indistinguishable from buffer-only controls. This pattern was consistent across all textile substrates. MC-nc27 showed negligible degradation activity on pure PET fiber, knit, and mesh textiles under both untreated and alkaline-pretreated conditions (Supplementary Figure S3), and on both cotton–PET blends (CP65/35 and CP55/45) regardless of pretreatment (Figure 4). Across every assay and substrate tested, MC-nc27 activity remained statistically indistinguishable from buffer-only background, consistent with a complete loss of catalytic function rather than a partial reduction in activity.

## 4 Discussion

Six *Tf*Cut2 variants were designed and evaluated across a series of substrates with increasing structural complexity: p-Nitrophenyl butyrate (pNPB), PET film, pure PET textiles, and cotton-PET blended textiles. Five of the variants produced measurable improvements in PET hydrolysis over the wild-type, while MC-nc27 was inactive across all substrates and conditions. A65H/L90A/I213S achieved the highest degradation activity at each PET substrate level. D12S and T234L performed consistently across both pure PET and blended textile formats. Fold improvements over wild-type ranged from approximately 1–2-fold on PET film to 8–14-fold on pretreated meshed PET textile. On cotton-PET blended textiles, where wild-type activity was near zero, A65H/L90A/I213S, D12S, and T234L reached A_260_ values of 1.043 ± 0.186, 0.961 ± 0.193, and 0.905 ± 0.192 on pretreated CP55/45, against −0.007 ± 0.033 for wild-type.

DSC confirmed that untreated knitted and mesh pure PET textiles had crystallinity values of 47.9% and 44.1%, respectively, both above the ∼30% threshold at which surface accessibility has been reported to become the dominant limitation on PET hydrolysis (Thomsen et al., 2022), providing a mechanistic basis for expecting that differences in crystallinity might differentially affect variants and alter their relative performance. Yet, although pretreatment reduced crystallinity to 22.1% and 22.7% in knitted and mesh textiles, respectively, a fixed-effects model testing the Treatment × Enzyme interaction on scaled performance found no evidence of hierarchy change across any substrate format (100% Fiber: F (4,30) = 0.936, *P* = 0.457; 100% PET Textile A: F (4,30) = 0.078, *P* = 0.989; 100% PET Textile B: F (4,30) = 0.168, *P* = 0.953). This test was restricted to the pure PET formats, for which crystallinity was measured before and after pretreatment (Table 3); it was not applied to the blends, whose crystallinity was not measured and whose cotton fraction adds a second pretreatment-sensitive variable. The low interaction F-values on both PET textile formats indicate that the hierarchy was nearly unchanged. Crystallinity is therefore unlikely to be the dominant variable governing relative variant performance. Instead, physical features of textiles that persist regardless of crystallinity state, such as fiber packing density, knit or mesh geometry, and interfiber occlusion, are the more probable constraints on differential enzyme-substrate engagement.

The pNPB and PET textile assays measure different aspects of enzyme activity. pNPB hydrolysis, conducted in homogeneous solution with a soluble substrate, primarily reports on active-site geometry and turnover under non-access-limited conditions, with an optimum near 35 °C, whereas PET film hydrolysis was favored at approximately 52 °C. A65H/L90A/I213 S exemplifies this discrepancy. For every other active variant, predicted pNPB activity was highest at the lowest temperature tested (35 °C), rather than at a defined interior optimum. A65H/L90A/I213 S was the exception, showing an interior local maximum at approximately 38 °C. Considered alone, this might suggest a distinct thermal preference. On PET film, however, A65H/L90A/I213S’s optimal temperature (50.9 °C) differed from wild-type (51.8 °C) by less than 1 °C, indicating that the modest temperature shift observed in the pNPB assay did not persist on PET film. The G62A/P193H/S197D and A65H/L90A/I213 S comparison shows a related discrepancy in relative ranking rather than thermal optimum. G62A/P193H/S197D produced the highest pNPB esterase signal of any construct yet ranked fourth or fifth on most textile substrates by A260, while A65H/L90A/I213S showed lower pNPB activity than G62A/P193H/S197D but achieved the highest A260 across every textile format. These observations extend prior cautions against proxy-substrate substitution (Pirillo et al., 2021; Wei et al., 2025), indicating that pNPB screening can misrepresent both the relative activity ranking of variants and their apparent thermal behavior on textile-grade PET. Examining performance across the substrate series therefore separates the variants into mechanistically interpretable groups.

The engineered *Tf*Cut2 variants demonstrated enhanced degradation performance across PET films, various forms of pure PET textiles, and meshed cotton-PET blended textiles with different PET contents. The characteristics and performance of each variant are discussed below.

A global docking search was used across all variants rather than a search restricted to the native active site, because the engineered substitutions target three structurally distinct regions: the native active site itself (A65H/L90A/I213S), a spatially separate designed auxiliary site (G62A/P193H/S197D), and a proposed surface-binding region outside the active site (H77R). Restricting the search to the native active site would have excluded the regions relevant to two of the three engineered hypotheses.

A65H/L90A/I213S showed the steepest performance gradient of any construct, increasing from 2.1-fold on PET film to 4.9-fold on pretreated PET fibers, 14.3-fold on pretreated mesh pure PET textile, and an A_260_ of 1.043 ± 0.186 on pretreated CP55/45, against −0.007 ± 0.033 for wild-type. L90A and I213 S were chosen by positional alignment with LCC to expand the substrate-binding groove, and Wei et al. (2016) reported that analogous substitutions in *Tf*Cut2 improve cutin hydrolysis. Mechanistically, these substitutions widen the hydrophobic binding groove that accommodates the apolar terephthalate rings of PET, reducing steric occlusion of the binding cleft so that more of the PET chain can be engaged, while A65H restores the binding affinity that L90A and I213S individually cost. Both substitutions individually reduce predicted binding affinity. A65H was selected by site-saturation mutagenesis to partially compensate for this loss, with molecular docking simulations scoring it at ΔΔG = −1.6 kcal/mol for structural stability. As this variant’s substitutions act directly at the native active site, its top-ranked docking pose was expected to localize there under the global search protocol described in the Methods. Molecular docking also predicted more productive docking positions for the triple mutation than for L90A/I213 S alone, consistent with partial active-site compensation by A65H. The performance gradient is consistent with this rationale, as groove expansion appears to pay off most where substrate access is most constrained, particularly under dense textile architecture and cotton fiber occlusion, where an unmodified binding cleft would limit productive surface engagement.

G62A/P193H/S197D activity scaled with substrate complexity but stayed below A65H/L90A/I213S at every level. This variant combines three substitutions. G62A has been reported to relieve MHET product inhibition and improve PET film degradation by 2.7- to 3.4-fold (Wei et al., 2016; Mrigwani et al., 2023). P193H and S197D were selected from the site-saturation screen to form a putative second Ser-His-Asp triad with the native Ser194, without compromising predicted PET-trimer binding. Because this designed auxiliary triad lies outside the native active site, a docking search restricted to the native site would not have captured ligand engagement at this region; the global search protocol was used specifically to evaluate binding at this spatially distinct site. Because G62A lies within the binding groove, it is expected to limit MHET rebinding, which keeps the site open for incoming PET chains and sustains turnover. On PET film, however, the variant improved on wild-type by only 1.03-fold, far below the reported gains. The benefit of G62A depends on relieving product inhibition, so its size should scale with MHET accumulation. The lower enzyme loading used here likely reduced turnover and MHET buildup, which would weaken this effect regardless of whether the mutation works as intended. G62A/P193H/S197D also produced the highest pNPB esterase signal of any active variant, well above wild-type. This may reflect a contribution of the engineered triad to the broader esterase activity of *Tf*Cut2, but single-mutant and pairwise controls would be needed to confirm it and to separate it from the effect of G62A alone. The pNPB advantage did not carry over to PET substrates: performance on untreated PET fiber and pretreated mesh textile improved on wild-type but did not track the esterase signal.

H77R/D204C/E253C was designed to pair a D204C/E253C disulfide bridge for calcium-independent thermostability (Then et al., 2016) with H77R, an SSM-predicted substitution outside the active site intended to improve binding through thermodynamic coupling with surface adsorption. This proposed surface-adsorption mechanism likewise falls outside the native active site, which was a second reason for using a global rather than active-site-restricted docking search. The optimal temperature on PET film was 51.8 °C, identical to wild-type, and calcium dependence was unaffected, suggesting the disulfide bridge did not form under BL21 (DE3) expression conditions, consistent with impaired disulfide formation in the reducing cytoplasm of standard *E. coli* strains. Despite this, the variant showed strong substrate-dependent scaling, with 1.18-fold improvement on PET film, 9.7-fold on mesh pure PET textile, and an A_260_ of 0.961 ± 0.193 on pretreated CP55/45. Given the absence of a detectable shift in optimal temperature, this gradient is unlikely to reflect thermostabilization and instead hints at H77R as the primary driver. On structurally complex textile surfaces, where productive enzyme-surface encounters are geometrically constrained, even a modest gain in surface-adsorption contacts from H77R’s increased binding affinity could account for the disproportionate performance gains observed at higher substrate complexity.

D12S and T234L each carry a single MutCompute-predicted substitution and both improved activity reproducibly across textile substrates. Optimal temperatures remained close to wild-type (52.7 °C and 53.2 °C versus 51.8 °C), pointing to improved structural robustness rather than altered thermal preference. By stabilizing the fold, these substitutions preserve the integrity of the shared hydrophobic groove and catalytic triad under prolonged near-Tg conditions rather than introducing new direct PET contacts, which is sufficient to enhance productive engagement of the substrate. Across both separated fibers and blended textiles, these variants consistently outperformed G62A/P193H/S197D and H77R/D204C/E253C, while remaining below A65H/L90A/I213S under the most complex substrate conditions. Notably, on pretreated CP55/45, D12S approached the performance of A65H/L90A/I213S (A_260_ = 0.961 versus 1.043), whereas wild-type activity remained near baseline. This indicates that a single stabilizing substitution can nearly match groove-engineering performance once alkaline pretreatment has reduced surface barriers. The gap between D12S and A65H/L90A/I213S widened on untreated knitted and mesh pure PET textiles, where intact fiber packing imposed the strongest constraints on enzyme-surface contact.

MC-nc27, which combined all non-conserved MutCompute-predicted substitutions, showed near-complete loss of activity across both pNPB and PET hydrolysis assays. This contrasts with the consistent gains produced by D12 S and T234L. The loss was substrate-independent and reproducible across expression batches, pointing to impaired folding or disrupted active-site geometry. This is consistent with the known non-additivity of multi-site mutational effects (Bell et al., 2022), and the result carries a practical implication. Simultaneous high-multiplicity mutagenesis is not a viable strategy in ML-guided enzyme engineering, even when individual predictions are sound. That D12 S and T234L performed well confirms the scoring function is not the problem. The failure is combinatorial.

Alkaline pretreatment with 15% NaOH at 80 °C for 4 h reduced crystallinity from 47.9% to 22.1% in knitted pure PET textile and from 44.1% to 22.7% in mesh pure PET textile, consistent with surface etching and partial saponification of accessible ester bonds that increase surface area (Čorak et al., 2022). A_260_ increased across all active constructs upon pretreatment. Wild-type *Tf*Cut2 showed no meaningful response, confirming that crystallinity reduction achieves little when the binding cleft cannot productively engage the textile surface, and that active-site engineering is necessary for hydrolytic activity on these substrates. Fold improvements over wild-type were nonetheless preserved within each substrate type whether pretreated or not, meaning the dominant performance differences across the substrate series reflect architecture rather than crystallinity state. A65H/L90A/I213S achieved the highest absolute pretreated activity despite showing the smallest proportional response to pretreatment, reflecting its elevated baseline, as the expanded groove already engages untreated textile more effectively than the unmodified binding cleft. Pretreatment opens surface access uniformly across active constructs, while groove geometry determines how many of those chains are converted to soluble products.

A65H/L90A/I213S also displayed a distinct pH profile relative to the other variants. At each variant’s own RSM-predicted optimal temperature, predicted activity at pH 8.5, expressed as a fraction of that variant’s peak activity, was 92.8% ± 5.3% for A65H/L90A/I213S, compared with 50.7%–79.2% for the other active variants (H77R/D204C/E253C: 50.7% ± 17.7%, D12S: 61.8% ± 12.9%, T234L: 71.3% ± 12.7%, wild-type: 73.8% ± 14.0%, G62A/P193H/S197D: 79.2% ± 11.5%; MC-nc27 excluded due to negligible, noise-dominated activity). This is potentially relevant to alkaline pretreatment, which leaves textiles at elevated pH prior to neutralization. However, the analysis is limited to PET film within the tested pH range (7.0–8.5), and whether tolerance extends above pH 8.5 or persists on pretreated textile substrates remains untested. This would need to be established before any conclusion regarding reduced neutralization requirements.

Several limitations warrant acknowledgment. The individual contribution of the auxiliary catalytic triad in G62A/P193H/S197D could not be isolated from that of G62A alone, as single-mutant and pairwise controls were not tested. The D204C/E253C disulfide bridge intended to raise the optimal temperature of H77R/D204C/E253C did not produce the expected shift, likely because disulfide bond formation is impaired in the reducing cytoplasm of standard *E. coli* expression strains. More broadly, the mechanistic basis for each variant’s activity was hypothesized from docking and MutCompute predictions alone, as molecular dynamics simulations, detailed structural interaction analysis of the enzyme-substrate complexes, and further docking validation were not performed. Protein purity was assessed only qualitatively, by Coomassie Blue staining and Western blot, rather than by quantitative densitometry. Though a pNPB-based correction factor is used to standardize batch-to-batch enzyme input, it does not substitute for direct purity quantification. Degradation was quantified throughout by absorbance at 260 nm, which captures the combined release of TPA, MHET, and BHET without distinguishing between them; consistent with prior absorbance-based PET-hydrolase assays (Zhong-Johnson et al., 2021; Thomsen et al., 2023a), A_260_ is treated as a relative rather than absolute measure of depolymerization. Because absorbance-based comparisons are less reliable for fold-differences below approximately 1.3-fold (Zhong-Johnson et al., 2021), the near-unity differences observed on PET film (for example, 1.03-fold for G62A/P193H/S197D and 1.18-fold for H77R/D204C/E253C) are not interpreted as meaningful activity differences; variant distinctions are instead drawn from the substantially larger fold-changes observed on textile substrates.

The pH tolerance observed for A65H/L90A/I213S was established on PET film within the tested range (pH 7.0–8.5), and whether it extends to higher pH or persists on alkaline-pretreated textile remains untested. Textile format coverage was also incomplete, as cotton-PET blends were tested only in mesh form. Finally, textile substrates were retail garments rather than genuine post-consumer waste; their blend compositions were taken from manufacturer specifications rather than independently verified, and neither their dye, finish, and additive content nor the fiber wear associated with prior use was characterized, leaving open how these factors may affect degradation in real-world waste streams.

Despite these limitations, the five active engineered variants demonstrated substantially enhanced PET degradation activity relative to wild-type *Tf*Cut2, with textile architecture appearing to be a major factor limiting wild-type degradation efficiency. The structural basis for each variant’s improvement remains unresolved and warrants further investigation. Priority directions include additional purification steps for enzyme loading, HPLC quantification of TPA, MHET, and BHET to replace A_260_ with absolute depolymerization rates (Pirillo et al., 2021), and direct confirmation of D204C/E253C disulfide bond formation or re-expression of H77R/D204C/E253C in a disulfide-permissive host such as SHuffle T7. Single-mutant and pairwise controls would help isolate the individual contributions of G62A and the auxiliary triad substitutions in G62A/P193H/S197D, and molecular dynamics simulations together with more detailed structural interaction analysis would help clarify the mechanistic basis of variant activity more broadly. Additional priorities include extending the A65H/L90A/I213S pH-tolerance analysis above pH 8.5 and onto pretreated textile, evaluating knitted cotton-PET blends, and characterizing and testing genuine post-consumer polycotton textiles, including their dye, finish, and additive content (Rosini et al., 2026). Across the substrate series, activity on textile-grade PET appears governed less by catalytic rate than by the capacity to engage a physically constrained, access-limited polymer surface, and that capacity is what future engineering of textile-active PET hydrolases should target.

## Data Availability

The original contributions presented in the study are included in the article/Supplementary Material, further inquiries can be directed to the corresponding author.
